# Crystalline heterogeneity in single ferroelectric nanocrystals revealed by polarized nonlinear microscopy

**DOI:** 10.1038/s41598-018-38229-4

**Published:** 2019-02-08

**Authors:** Carolina Rendón-Barraza, Flavia Timpu, Rachel Grange, Sophie Brasselet

**Affiliations:** 1 Aix Marseille Univ, CNRS, Centrale Marseille, Institut Fresnel, F-13013 Marseille, France; 2 Optical Nanomaterial Group, Institute for Quantum Electronics, Department of Physics, ETH Zurich, Auguste-Piccard-Hof 1, 8093 Zurich, Switzerland; 3 Present Address: Ultrafast and Microspectroscopy Laboratories and ARC Centre of Excellence in Exciton Science, School of Chemistry, University of Melbourne, Melbourne, 3010 Australia

## Abstract

Ferroelectric nanocrystals have considerable interest for applications in nanophotonics, optical memories and bio-imaging. Their crystalline nature at the nanoscale remains however poorly known, mostly because structural investigation tools on single nanocrystals are lacking. In this work we apply polarization resolved second harmonic generation (P-SHG) imaging on isolated Barium Titanate (BaTiO_3_) nanocrystals to unravel their crystalline nature, exploiting the sensitivity of polarized SHG to local non-centrosymmetry and nanocrystals surface responses. We evidence crystalline heterogeneities in BaTiO_3_ nanocrystals manifested by a centrosymmetric shell around the tetragonal core of the crystals, corroborating hypotheses from previous ensemble structural investigations. This study shows that in contrast to bulk materials, nanocrystals exhibit a complex composition, which is seen to be reproducible among nanocrystals. P-SHG appears furthermore as a powerful methodology that reports structural behaviors in nanoscale dielectrics materials, at the individual nanoparticle scale.

## Introduction

Understanding the optical and physical processes occurring at the nanoscale in nanomaterials is fundamental for various applications that cover their use as nano-emitters in imaging, as ferroelectric nano-objects in non-volatile memories and piezo-electric devices, but also for electro-optics and more generally nanophotonics. One of the most attractive property of dielectric and ferroelectric nanocrystals is their ability to generate efficient nonlinear optical interactions even though their size scale down below hundreds of nanometers, which opens to an extensive range of functions that rely on optical frequency mixing. Barium Titanate (BaTiO_3_) nanocrystals are particularly interesting in this prospect, being ferroelectric oxides that exhibit a stable tetragonal phase at room temperature. BaTiO_3_ nanocrystals are thus efficient non-centrosymmetric second harmonic generation (SHG) emitters, which signal is detectable down to 20 nm sizes^1^, making them suitable biomarkers for nonlinear background-free imaging in cells or tissues^2,3^. Recently the SHG efficiency of BaTiO_3_ nanocrystals has been even further improved by plasmonic enhancement^4^ or benefiting from Mie resonances^5^. BaTiO_3_ nanocrystals are thus promising candidates for nonlinear imaging, along with other oxides and dielectric crystals^6–8^.

Despite their recognized efficiency, BaTiO_3_ nanocrystals exhibit complex structural behaviors that are still under debate^9,10^. Bulk BaTiO_3_ crystals are known to undergo a transformation from a cubic into a tetragonal structure below 130 °C, further followed by an orthorhombic form below 0 °C^11^. Under different constraint such as varied chemical preparation conditions, strain, or at small sizes, BaTiO_3_ is however prone to morphotropic phase boundary, which allows the co-existence of competing crystalline phases. In particular, transition from tetragonal to cubic phases below a critical particle size of about 50 nm has been observed^12,13^, as well as the formation of a disordered shell around a tetragonal core^14,15^. Studies on powder X-ray diffraction have confirmed this view by revealing that the surface of BaTiO_3_ nanocrystals relaxes to the cubic paraelectric phase, with an increasing contribution at small nanocrystal sizes^16^. Such phases have however never been directly imaged at the single nano-object scale by lack of measurable nanoscale properties, leaving many unknowns on the composition of individual nanocrystals and its heterogeneity among nanocrystals.

As a consequence, there is today a poor knowledge on how the structure of such nanocrystals influences their optical functions, which are averaged over the scale of optical diffraction limit (e.g. a few hundreds of nanometers). This knowledge is yet a key element for the design of future nanophotonics devices. In this work, we address the question of the structure and crystalline heterogeneity of BaTiO_3_ nanocrystals by the implementation of a direct optical method that is able to reveal structural features at scales smaller than the diffraction limit. This method is based on polarization resolved second harmonic generation (P-SHG) imaging, a process that relies on the sensitivity of SHG to the incident polarization at each measured point of the nanocrystal.

**Figure 1:**
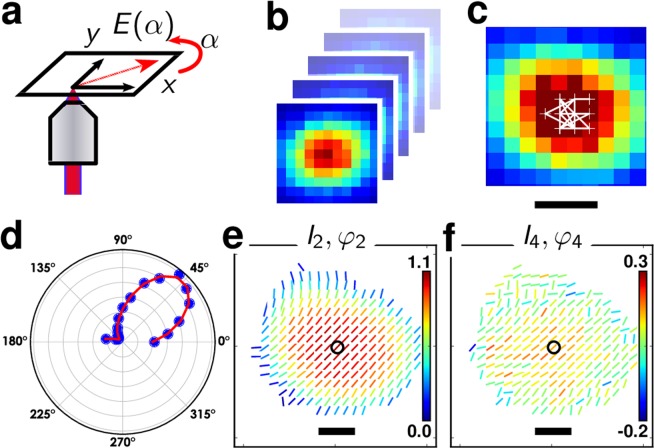
(**a**) Principle of P-SHG measurement method, depicting the polarization angle *α* which rotates over [0°–180°]. (**b**) A SHG images stack is formed by raster scanning the sample for each *α*. The data are shown for a KTP nanocrystal of 150 nm size. Pixel size: 50 nm. (**c**) Calculated centroids (in white) for each image of a P-SHG stack. (**d**) SHG intensity as a function of α, in polar representation, here shown for the pixel in the center of (**c**). (**e**) P-SHG analysis results. The stick orientation represents the orientation *φ*_2_ obtained for each pixel, while its color is encoding the *I*_2_ value for this pixel. (**f**) Corresponding (*I*_4_, *φ*_4_) map for the same KTP nanocrystal, using similar representation for the stick orientation (*φ*_4_) and color (*I*_4_). Scale bars: 200 nm.

The light-matter coupling process at the origin of SHG nonlinear radiation in crystals is intrinsically vectorial. It strongly depends on the orientation of the crystal, the symmetry of its crystalline unit cell, and the polarization direction of the incident electromagnetic field^17^. The principle of P-SHG imaging, schematically depicted in Fig. 1a, takes advantage of this vectorial coupling to probe spatially the nature of the crystal orientation and unit cell symmetry, by a modulation of the incident light polarization direction at each pixel of a scanning microscope. In contrast to averaging the nonlinear polarized responses from each nanocrystal^18–21^, this method thus expands spatially the monitoring of SHG polarized responses. By adding an extra degree of freedom (polarization) to spatial scanning, P-SHG allows to probe the structure of nano-objects in their spatial dimension. This method has recently permitted to map nonlinear vectorial coupling properties at the surface of metallic nanoparticles of complex shapes, which plasmonic modes where spatially extended and strongly anisotropic^22^. In the present work, the sample is made of isolated dielectric nanocrystals deposited on a microscope cover slip from a diluted solution. Mapping spatial heterogeneities in dielectric nanoparticles is complexified by their unknown orientations, in contrast to pre-defined metallic nanostructures fabricated by nanolithography. Nevertheless we show in this work that the rich nature of polarized SHG added with imaging capabilities is capable of extracting structural information even in such situation. The samples used in this work are either made of KTiOPO_4_ (KTP) of 150 nm size used as a reference^7,23^, or BaTiO_3_ (named BTO in what follows) of 100 nm size, unless otherwise mentioned (see Methods).

## Results and Discussion

### Principle of P-SHG imaging

SHG images are formed by a point scanning two-photon excitation microscope at 800 nm incident wavelength, in which the linear polarization of the excitation beam is controlled in the sample plane (see Methods). For each nanocrystal, a stack of SHG images is formed that contains typically 18 images, corresponding to polarization angles α ranging regularly from 0° (with respect to the horizontal direction) to 162° (Fig. 1b). In order to access sub-diffraction pixel size accuracy, images are corrected for lateral spatial drift that arises from mechanical drift and slight spot position motion during the course of the half wave plate rotation that ensures polarization tuning (Fig. 1c) (see Methods). Each pixel of the obtained image stack thus contains a modulated SHG intensity I(*α*), called P-SHG response, which is the result of the coupling between the rotating incident polarization and the nanocrystal portion illuminated by the beam parked at the pixel position (Fig. 1d). Averaging over typically 8 measurements permits to further increase the signal-to-noise ratio.

P-SHG intensities I(*α*) obtained at each pixel of the drift-corrected, averaged nanocrystal image are then decomposed into a Fourier series. Since SHG responses depend on the fourth power of the in-plane incident field *E*(*α*), I(*α*) can be decomposed onto circular functions of second and fourth orders^17,22^:

1 $${\rm{I}}(\alpha ){/I}_{0}\propto 1+{a}_{2}\,\cos \,2\alpha +{b}_{2}\,\sin \,2\alpha +{a}_{4}\,\cos \,4\alpha +{b}_{4}\,\sin \,4\alpha $$

Where I_0_ is the total intensity, resulting from the sum over all incident polarization angles. Here the incident field polarization is assumed to be rotating in 2D with coordinates in the sample frame $E(\alpha )=(\cos \,{\rm{\alpha }},\,\sin \,\alpha ,0)$. In a first approximation the field longitudinal contribution is neglected, due to its low magnitude in comparison to its in-plane components^24^. This approximation is discussed below. The (*a*_2_, *b*_2_, *a*_4_, *b*_4_) coefficients are retrieved experimentally by projection on circular functions, following an approach previously described^22^. They can be grouped into amplitude and phase terms which reflect the second and fourth orders of the *α*-dependence of the P-SHG response:

2 $$\begin{array}{ll}{I}_{2}=\,\sqrt{{a}_{2}^{2}+{b}_{2}^{2}}, & {I}_{4}=\varepsilon \sqrt{{a}_{4}^{2}+{b}_{4}^{2}}\\ {\phi }_{2}=\,\,\frac{1}{2}\arctan \frac{{b}_{2}}{{a}_{2}}, & {\phi }_{4}=\frac{1}{4}\arctan \frac{{b}_{4}}{{a}_{4}}\end{array}$$

with *φ*_4_ given modulo π/2. $\varepsilon =\,\cos \,4({\phi }_{4}-{\phi }_{2})\,$ sets the sign of I_4_ such that the fourth order response lies either along the second order (*I*_4_ > 0) response or π/4 phase shifted (I_4_ < 0)^22,25^.

Altogether, (*I*_2_, *I*_4_) and (*φ*_2_, *φ*_4_) are direct signatures of the shape (modulation amplitude, phase) of the P-SHG modulation response present at each pixel position (see Fig. S1). *I*_2_ reports its local anisotropy, e.g. the amplitude of its low-order modulation, while *I*_4_ represents the amplitude of its higher order response. Physically, these parameters reflect the symmetry of the vectorial coupling that locally takes place in the nanocrystal at the spotted pixel position, as a result of its specific unit-cell symmetry and orientation projected in the sample plane. A structure with strong dipolar symmetry will yield a dominant *I*_2_ value, while a multipolar symmetry structure is likely to lead to a weaker *I*_2_ and a stronger *I*_4_ contribution. (*φ*_2_, *φ*_4_) on the other side represent the in-plane orientations of these responses, e.g. the mean direction of the anisotropy (*φ*_2_) and fourth order response (*φ*_4_). (*φ*_2_, *φ*_4_) are thus directly linked to the orientation of the unit-cell structure probed at the specific pixel position. The P-SHG analysis results are rendered as oversampled images, using a pixel size of 50 nm (Fig. 1e,f). Each pixel is represented by a stick which orientation points the *φ*_2_ (resp. *φ*_4_) direction, and which color is encoded with the *I*_2_ (resp. *I*_4_) values. The signal level used for P-SHG analysis is thresholded with a lower limit that ensures standard deviations lower than 0.01 on the (*I*_2_, *I*_4_) parameters, accounting moreover for a pre-calibrated bias that occurs at low signal to noise conditions^22^.

Figure 1e,f shows the result of a P-SHG analysis on an isolated 150 nm size KTP nanocrystal. KTP exhibits a unit-cell which, when the main optical axis lies in the sample plane, is dominated by a single direction that makes it a close-to-dipolar response^26^. This is consistent with the (*I*_2_, *φ*_2_) map recorded for the KTP nanocrystal (Fig. 1e) which is visibly that of such a strongly anisotropic structure, oriented along *φ*_2_ in the sample plane, with *I*_2_ values homogeneously distributed within its volume. Similarly, the (*I*_4_, *φ*_4_) map (Fig. 1f) shows low *I*_4_ values oriented along the same direction as *φ*_2_. The P-SHG maps exhibit a larger spatial extent than the pure intensity SHG image, which is due to the large persistence of the polarization modulation signal even at low intensity values, as reported in^22^. It thus potentially reveals information at the sub-diffraction scale about the orientational organization of nonlinear induced dipoles within this focal spot.

### P-SHG imaging of BaTiO_3_ nanocrystals

**Figure 2:**
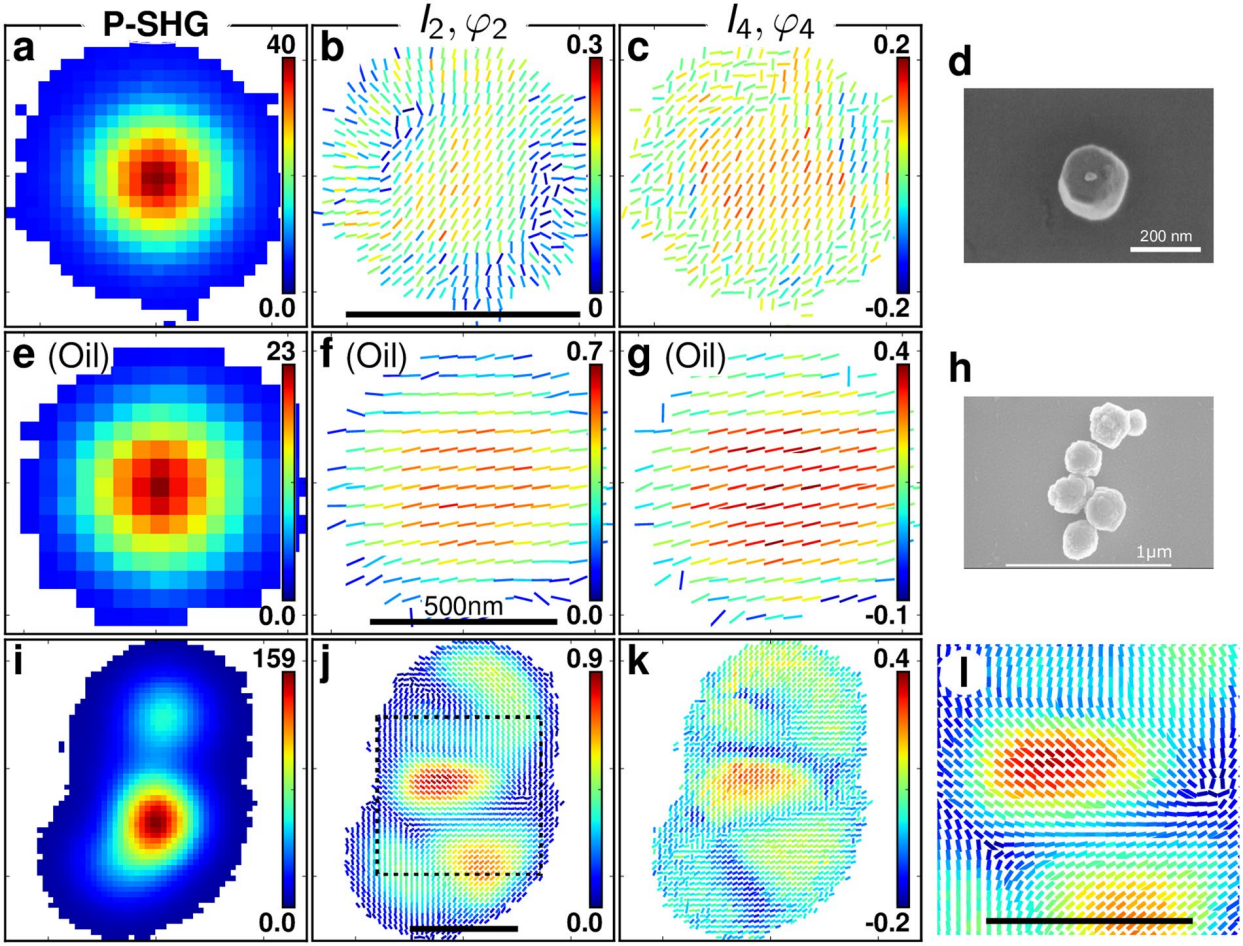
(**a**) Total SHG intensity image of a BTO nanocrystal of size ~200 nm (pixel size: 50 nm). (**b**) Corresponding P-SHG result showing *I*_2_ (as the stick color encoding), *φ*_2_ (as the stick orientation) per measured pixel. (**c**) P-SHG results showing (*I*_4_, *φ*_4_). (**d**) SEM image of this BTO nanocrystal. (**e**) P-SHG total intensity image of a 100 nm size BTO nanocrystal covered with oil. (**f**) Corresponding (*I*_2_, *φ*_2_) and (**g**) (*I*_4_, *φ*_4_) maps. (**h**) SEM image of a ~ 1000 nm size aggregate of BTO nanocrystals. (**i**) P-SHG total intensity image of this aggregate. (**j**) Corresponding (*I*_2_, *φ*_2_) and (k) (*I*_4_, *φ*_4_) maps. (**l**) Enlarged view of the highlighted area in (**j**). Scale bars: 1000 nm unless otherwise noted.

Figure 2a–c shows typical P-SHG results obtained from a single BaTiO_3_ (BTO) nanocrystal of about 200 nm size, which has been imaged in parallel by scanning electron microscopy (SEM) (Fig. 2d) (see Methods). While the pure intensity SHG image defined by the total intensity *I*_0_ is a Gaussian-like shape characteristics of the imaging point spread function (PSF) convolved by the size of the nanocrystal (Fig. 2a), its P-SHG maps depict very different behaviors. First, (*I*_2_, *I*_4_) values are not uniformly distributed over the size of the P-SHG image, in contrast to what is observed in KTP nanocrystals. Second, *φ*_2_ and *φ*_4_ values, represented by (*I*_2_, *I*_4_) sticks orientations, exhibit strong differences between the center of the map, where these angles are uniform, and the border, where they are radial-like (note that *φ*_4_ is determined modulo *π*/2, leading to equivalent angles between *φ*_4_ and *φ*_4_ + *π*/2). The shapes of nanocrystals are not perfectly spherical, as depicted in the SEM image (Fig. 2d), however this strong heterogeneity cannot occur solely due to shape imperfections since center and border responses strongly differ. The radial pattern rather seems to originate from a strong anisotropy value of *I*_2_ along the normal direction to the surface of the structure.

Since this behavior resembles a surface-like SHG response as already observed in metallic nanoparticles^22^, we tested this hypothesis by changing the surface index mismatch of the nanocrystal. Remarkably, embedding the BTO nanocrystals into a high index medium (oil immersion) cancels this radial pattern and leads to homogeneous (*I*_2_, *φ*_2_) and (*I*_4_, *φ*_4_) maps that behave now similarly to KTP nanocrystals (Fig. 2e–g). The average refractive index of BTO (n = 2.45 at 800 nm)^27^ is higher than for KTP (n = 1.75 at 800 nm)^28^, thus BTO exhibits a higher surface index mismatch in air and is more likely to create surface induced responses from a stronger local normal asymmetry^29^. This surface response has been identified in early works on SHG in isotropic media^30,31^, and has be shown to be non-negligible in centrosymmetric media, in particular crystals^32–35^. It can be described as an effective *χ*^(2)^ susceptibility tensor which includes dipolar and multipolar contributions arising from both the structural discontinuity that breaks the centrosymmetry at the crystal interface and the strong local fields gradients^31^. The associated tensor exhibits three independent components: ${\chi }_{ttn}^{(2)},{\chi }_{ntt}^{(2)}$ and ${\chi }_{nnn}^{(2)}$, where *n* is the surface normal direction and *t* denotes directions parallel to the surface^36^. The diagonal element ${\chi }_{nnn}^{(2)}$ has been estimated to be at least one order of magnitude stronger than the other components, leading to a dominant nonlinear induced polarization oriented normal to the surface^32–34^. Additionally in BaTiO_3_ nanoparticles, the incident field locally scattered by the nanoparticle has a specific geometry. Finite element method simulations^5^ show that at 800 nm illumination, the local field exhibits strong gradients at the interface with a preferred polarization along the normal direction (see Fig. S2). Remarkably, the field magnitude at the interface in air for a 100 nm size particle is about five times stronger than at the nanoparticle center (see Fig. S2). This scattering efficiency is expected to decrease when the index of the surrounding environment approaches that of the nanocrystal. All those elements favor a privileged nonlinear coupling along the normal direction at the nanocrystal interface, together with strong surface nonlinearities. Note that inside the nanoparticle, the local field polarization follows the incident polarization direction (Fig. S2).

Overall, the P-SHG pattern observed in Fig. 2b,c could thus occur from two contributions: a central oriented pattern coming from the bulk non-centrosymmetric unit cell of the crystal, and a radial pattern coming from the surface contribution representative of a dipolar response in the normal direction to the local crystal-air interface.

Interestingly, the P-SHG approach used here permits to visualize those contributions distinctively, thanks to the spatial scanning and oversampling of the polarization analysis. This effect is invisible from more traditional polarization SHG analyzes that average the signal over the whole particle when focused at its center^18,19,37^. This approach permits to evidence other features: occasionally nanocrystals aggregates could be found on the sample surface, which is indicated by a broader SHG intensity image (Fig. 2i). This image is however unable to inform about the number and arrangement of the nanocrystals within the aggregate. Observing the P-SHG maps (Fig. 2j–l), in contrast, provides this hidden information by clearly separating structures of different orientations. Figure 2j,k depicts at least five different sub-units that also appear in the SEM picture of the same aggregate, while the pure SHG image (Fig. 2i) shows only two large spots.

### Modelling of P-SHG images in BaTiO_3_

**Figure 3:**
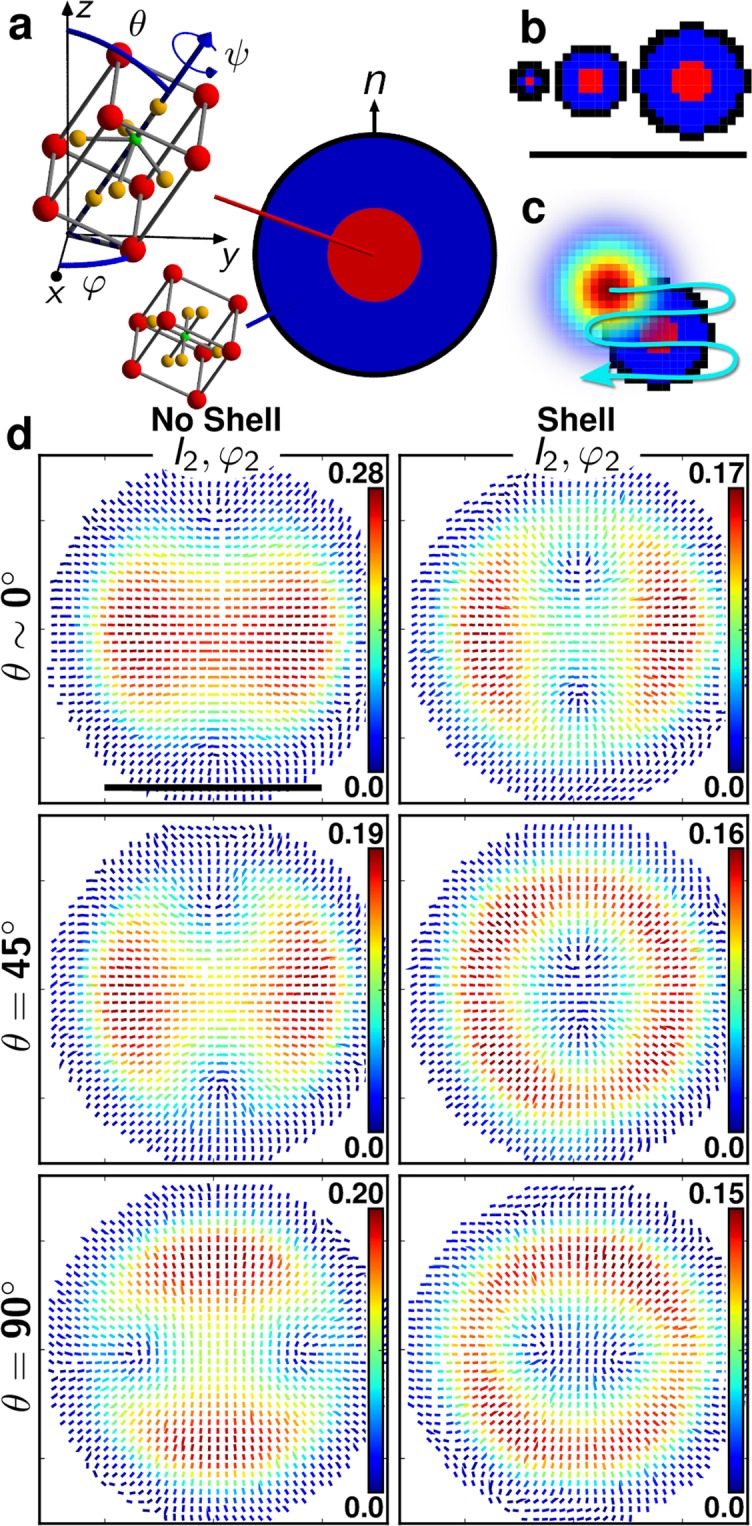
(**a**) Schematic representation of a BTO nanocrystal modelled as a 2D disk of tetragonal symmetry (in red) embedded in a cubic shell (in blue). The tetragonal crystal is of *4 mm* symmetry class and its main axis orientation is denoted by the polar angles (*θ*, *φ*, *ψ*). The cubic crystal is centro-symmetric (*m3m* symmetry class) and has no SHG response. The schematic representation of the corresponding unit cell atoms are colored in red for barium, green for titanium, and orange for oxygen. The interface (black rim) is of 1 pixel thickness (1 px = 40 nm) and modelled as nonlinear 1D dipoles oriented along the surface normal (*n*). (**b**) Representation of three diameter sizes: 100 nm (2.5 px), 300 nm (7.5 px) and 500 nm (15 px), with an identical core-to-size ratio *r* = 0.4 (*r* = *c*/*s* with *c* the core diameter and *s* the total diameter size of the nanocrystal). (**c**) Schematic representation of the 2D Gaussian optical focus (250 nm size) scanned to simulate diffraction-limited P-SHG imaging. (**d**) Resulting theoretical P-SHG images for a 100 nm size nanocrystal, at different *θ* angles, without the shell (left) and in presence of the shell (right, using *r* = 0.4). Scale bars: 1000 nm. The surface-to-core nonlinear efficiency ratio is *η* = 0.5.

To understand the radial features appearing in Fig. 2b,c, we modelled the nanocrystal by its unit-cell tetragonal symmetry characteristics of bulk BTO^38^, in presence of a surface contribution. The model structure is represented in Fig. 3a, where the nanocrystal is made of a core of tetragonal symmetry and a possible surface contribution along its local normal direction *n*. We additionally allowed the existence of a shell between the core and the interface, which structure is centrosymmetric, similarly to results previously obtained from X-ray diffraction on BTO nanocrystal powders^16^. This study conjectured an intermediate gradient structure between the core and the shell which is ignored here for simplicity. In this model, the nanocrystal is simplified by a 2D disk illuminated by a focused electric field which polarization rotates in the sample plane. This approximation supposes first that the longitudinal nonlinear coupling is negligible. Under the used numerical aperture (NA = 1.15), the longitudinal field amount is about 33% of its in-plane components (on average over its point spread function), which leads to bias of P-SHG results by no more than 20% in BTO (see Fig. S3). Note that the effect of this contribution gets stronger when the crystal’s main symmetry axis lies along the propagation direction, and when the crystal is more one-dimensional, such as for KTP^39^ (see Fig. S3). Second, the expansion of the particle shape in 3D is not expected to strongly affect the P-SHG response. Indeed the nanocrystal bulk core response would lead to unchanged P-SHG response, while the surface response occurring from the upper and lower parts of the sphere would lead to a pure longitudinal coupling which is isotropic in the sample plane and thus vanishing for P-SHG.

In the model, a Gaussian spot of size 250 nm scans the circular nanocrystal (Fig. 3b), which is sampled with local nonlinear response tensors *χ*^(2)^ positioned on a grid every 40 nm steps (see Supplementary Information). *χ*^(2)^ is defined inside the core of the nanocrystal by the BTO crystalline tetragonal symmetry ${\chi }_{BTO}^{(2)}$, using coefficients found in^40^, in the shell by a cubic symmetry for which *χ*^(2)^ = 0, and at the surface by a unique coefficient ${\chi }_{nnn}^{(2)}\,$ with *n* the surface normal direction. The relative efficiencies between the core and surface nonlinearities is expressed by a ratio $\eta =\Vert {\chi }_{nnn}^{(2)}\Vert /\Vert {\chi }_{BTO}^{(2)}\Vert$, with $\Vert \Vert$ the tensorial norm. This tensor is moreover rotated using the Euler set of angles (*θ*, *φ*, *ψ*) (Fig. 3a)^25^. The structure expands over different sizes, from 50 nm to 300 nm with identical core-to-size ratios *r* (Fig. 3b). For each position of the Gaussian spot, and for each incident polarization angle *α*, the nonlinear intensity is computed from the coherent addition of radiations from all nonlinear dipoles covered by the spot (Fig. 3c, see Supplementary Information) and analyzed using Eq. (1). This response is finally rendered as a (*I*_2_, *φ*_2_) map illustrated in Fig. 3d for different nanocrystal structures (in presence or not of a shell) and 3D orientations. Only *θ* is varied, since (*φ*, *ψ*) involve only a rotation of the P-SHG maps rather than a pattern change, due to the symmetry of the nanocrystal (Fig. 3a). Note that those responses are not considerably modified when the model accounts for a field longitudinal contribution in 3D (see Fig. S4).

Interestingly, modelling a pure tetragonal symmetry surrounded by a surface response (Fig. 3d, left) leads to very different responses than those obtained for a structure made of a tetragonal core, a centrosymmetric shell and a surface response (Fig. 3d, right). In particular, radial features in the (*I*_2_, *φ*_2_) maps appear dominantly in the presence of a shell whatever the nanocrystal orientation, size or *η* ratio (see Figs S5–S8 for *η* = 0.5 and Figs S9–S12 for *η* = 1). By introducing a centrosymmetric shell in the structure, the radial surface response originating from the nanocrystal surface discontinuity is visibly emphasized, evidencing both the presence of the shell and the surface response. Figure 3 finally shows that the presence of an interface response is essential to reveal the existence of a centrosymmetric shell in the nanocrystal structure.

At low *θ* angles in the presence of a shell, the (*I*_2_, *φ*_2_) maps depart from a pure radial shape and take a more unidirectional feature (Fig. 3d, upper right). This corresponds to the situation where the optical axis of the BTO tetragonal core is almost along the propagation direction, for which even a small tilt off plane produces a dipolar-like P-SHG response. Note that such unidirectional responses are also observed for other *θ* angles in cases where the nanocrystal size is much lower than 100 nm (see Fig. S5), which is not the size range investigated here. At last, increasing the interface strengths (*η *≥ 1) tends to induce purely radial-like patterns whatever the *θ* value with a vanishing *I*_2_ at the center (see Figs S9 to S12). Importantly, situations that can appear similar in (*I*_2_, *φ*_2_) patterns can differ in (*I*_4_, *φ*_4_) responses. Consequently, to discriminate the presence or not of a shell and the interface nonlinear strength contribution, both patterns need to be compared to models.

### Interpreting experimental P-SHG results in BaTiO_3_ nanocrystals

**Figure 4:**
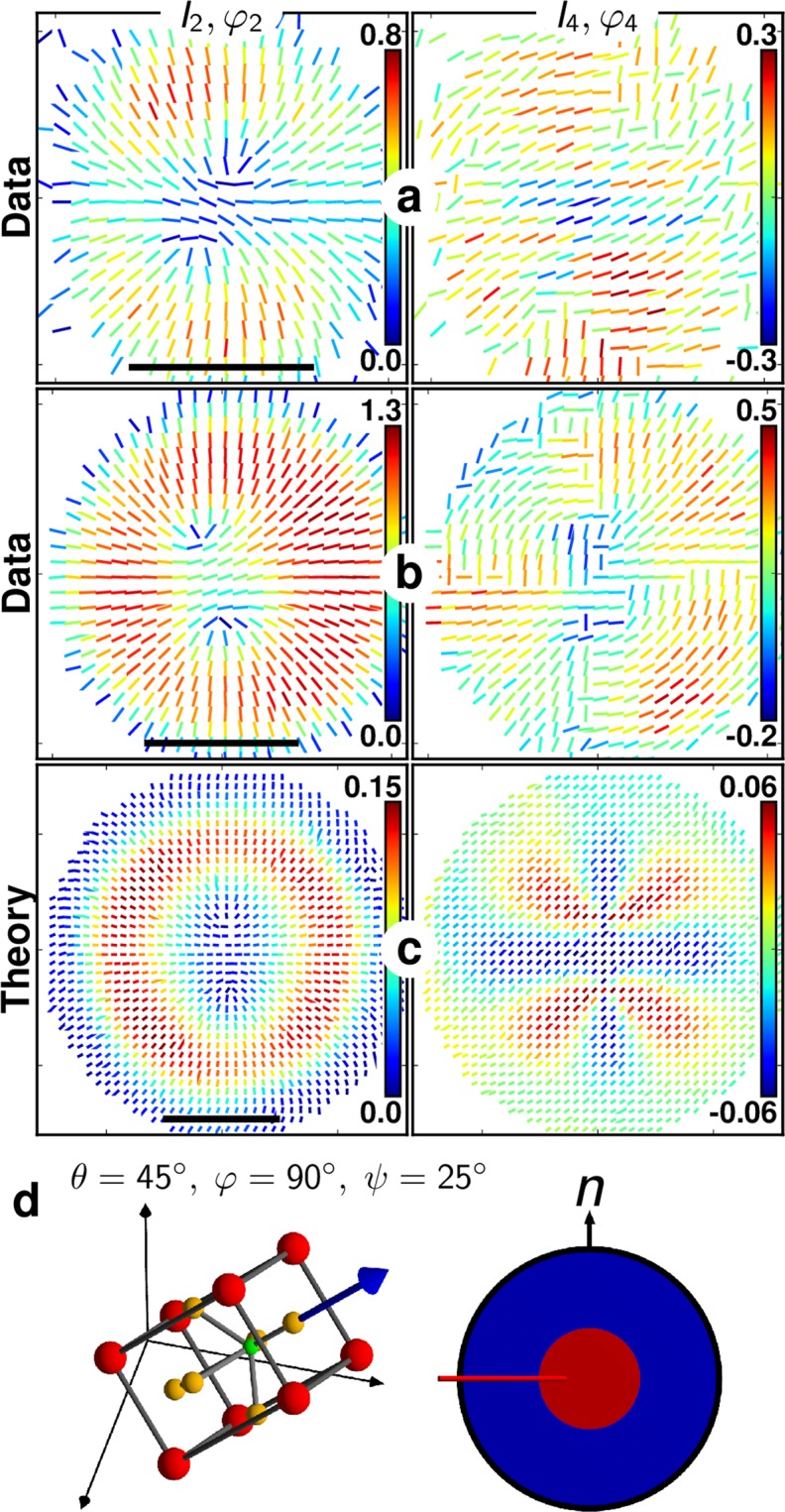
(**a**,**b**) Second- and fourth-order P-SHG experimental results for two measured BTO nanoparticles and (**c**) one modelled nanoparticle. Scale bars: 500 nm (12.5 px). (**d**) The model parameters are *θ* = 45° (variable *φ* and *ψ* yield identical results), nanocrystal diameter 100 nm (2.5 px), core-to-size ratio *r* = 0.4, surface to core nonlinearity ratio *η* = 0.5.

Figure 4 depicts typical radial-like (*I*_2_, *φ*_2_) and (*I*_4_, *φ*_4_) responses measured in part of the BTO nanocrystals. This population corresponds to 25% of the 60 measured nanocrystals. Comparing qualitatively the experimental patterns with theoretical responses for both second and fourth orders in 100 nm size nanocrystals permits to deduce that they exhibit a cubic shell of core-to-size ratio around *r* = 0.4 and a non-negligible surface contribution of *η* = 0.5. A typical range of *η* that leads to a reasonably good agreement with the experimental observations is [0.2–0.7]. Note that we do not expect an exact quantitative correspondence between experiment and theory, since the nanocrystal is simulated in 2D, using a simplified structural model, and the incident field is supposed spatially homogeneous without any longitudinal contribution, which is a first order approximation. Nevertheless, this comparison permits to reveal the structural heterogeneity of such nanocrystals, to give order of magnitudes of its spatial extent, and to estimate its 3D orientation. In particular, Fig. 4a is likely to correspond to a nanocrystal for which *θ* lies between 0° and 45° (a good agreement is found for *θ* ~ 40°), while Fig. 4b resembles more an orientation *θ* higher than 45°. *ψ* does not have a major effect on the P-SHG patterns, due to the tetragonal symmetry structure, while *φ* governs the in-plane orientation of the P-SHG pattern only for *θ* < 45°.

Notably, the patterns observed in Fig. 4 are a clear indication of the existence of a centrosymmetric shell around the tetragonal core in the nanocrystal. Note that the experimental results shown in Fig. 2b,c exhibit slightly different features for which (*I*_2_, *I*_4_) values in the central part of the patterns are not vanishing. This case is found to correspond to a larger nanocrystal size of about 300 nm diameter, with a core-to-size ratio *r* = 0.4 and *η* = 0.5 (see Fig. S4 for comparison with the model). This result is consistent with the larger particle size observed in Fig. 2b as compared to Fig. 4.

**Figure 5:**
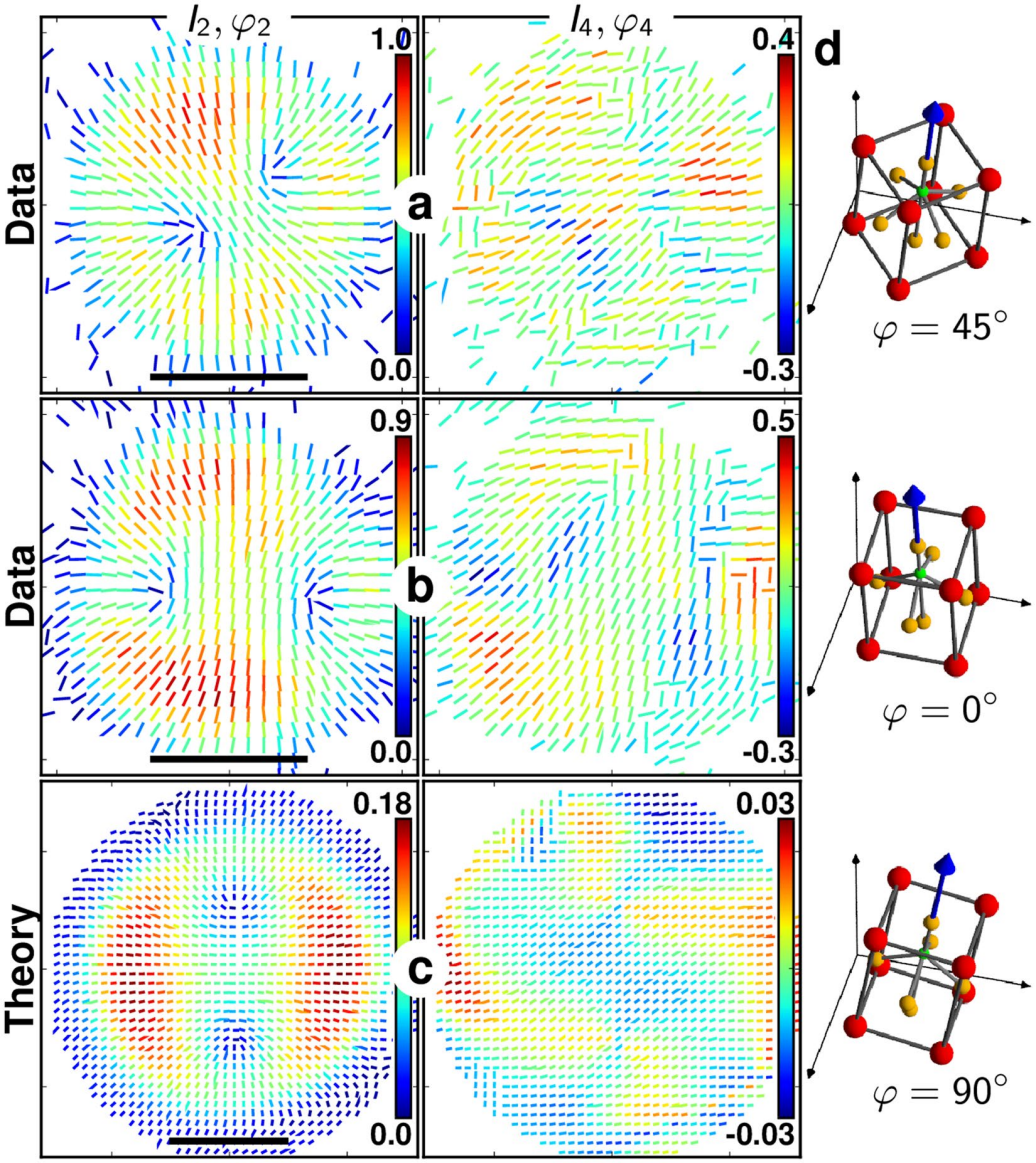
(**a**,**b**) Second- and fourth-order P-SHG results for two measured BTO nanoparticles and (**c**) one modelled nanoparticle (size 100 nm, *r* = 0.4, *η* = 0.5, *θ* ~ 0°, *φ* = 90°). Scale bars: 500 nm. (**d**) The suggested corresponding crystal orientations (for a,b, and c, respectively) are *θ* ~ 0° with *φ* = 45°, 0°, 90°.

Among the rest of the BTO nanocrystals measured, features appear that differ slightly from a pure radial response (Fig. 5, see other examples in Fig. S13). Such responses are more ambiguous since they could be found in the model from both shell (for *θ* < 45°, as shown in Fig. 3d, right) and non-shell structures (Fig. 3d, left). To differentiate the presence or not of a shell in ambiguous (*I*_2_, *φ*_2_) patterns, it is necessary to investigate more closely the fourth order symmetry (*I*_4_, *φ*_4_) response of such nanocrystals (see Fig. S6). In particular, the center of the (*I*_4_, *φ*_4_) pattern is non-vanishing in the presence of the shell while with no shell, it completely vanishes (see Fig. S6). From a comparison of (*I*_4_, *φ*_4_) patterns, we conclude that the observations of Fig. 5 are representative of nanocrystals of similar composition as above (Fig. 4), but oriented with their main crystalline axis close to the longitudinal direction (i.e. *θ* ~ 0°). Interestingly, the anisotropy of this pattern permits moreover to identify the azimuthal in-plane orientation of the nanocrystal, as illustrated in Fig. 5d. Overall these anisotropic patterns were observed in 30% of the measured nanocrystals. The rest of the observed patterns depicted non-negligible central *I*_2_ and *I*_4_ values such as in Fig. 2b (see also Fig. S14), which we attribute to nanocrystals of larger sizes with similar composition.

Finally, the different cases investigated by P-SHG evidence the presence of a centro-symmetric shell around the tetragonal core of BaTiO_3_ nanocrystal, which size is of the order of 40% of its total diameter, representing tens of nanometers. This nano-scale structural information is obtained by the analysis of spatial polarization modulation dependences, together with an a priori knowledge of the nanocrystal structure. In this respect, P-SHG is different from a super resolution imaging method, since it does not directly reconstruct a crystalline nano-scale picture. Nevertheless, SHG polarization responses from known nanocrystals can serve as markers for super resolution imaging^41^.

### Quantitative estimation of BaTiO_3_ nanocrystals orientations

**Figure 6:**
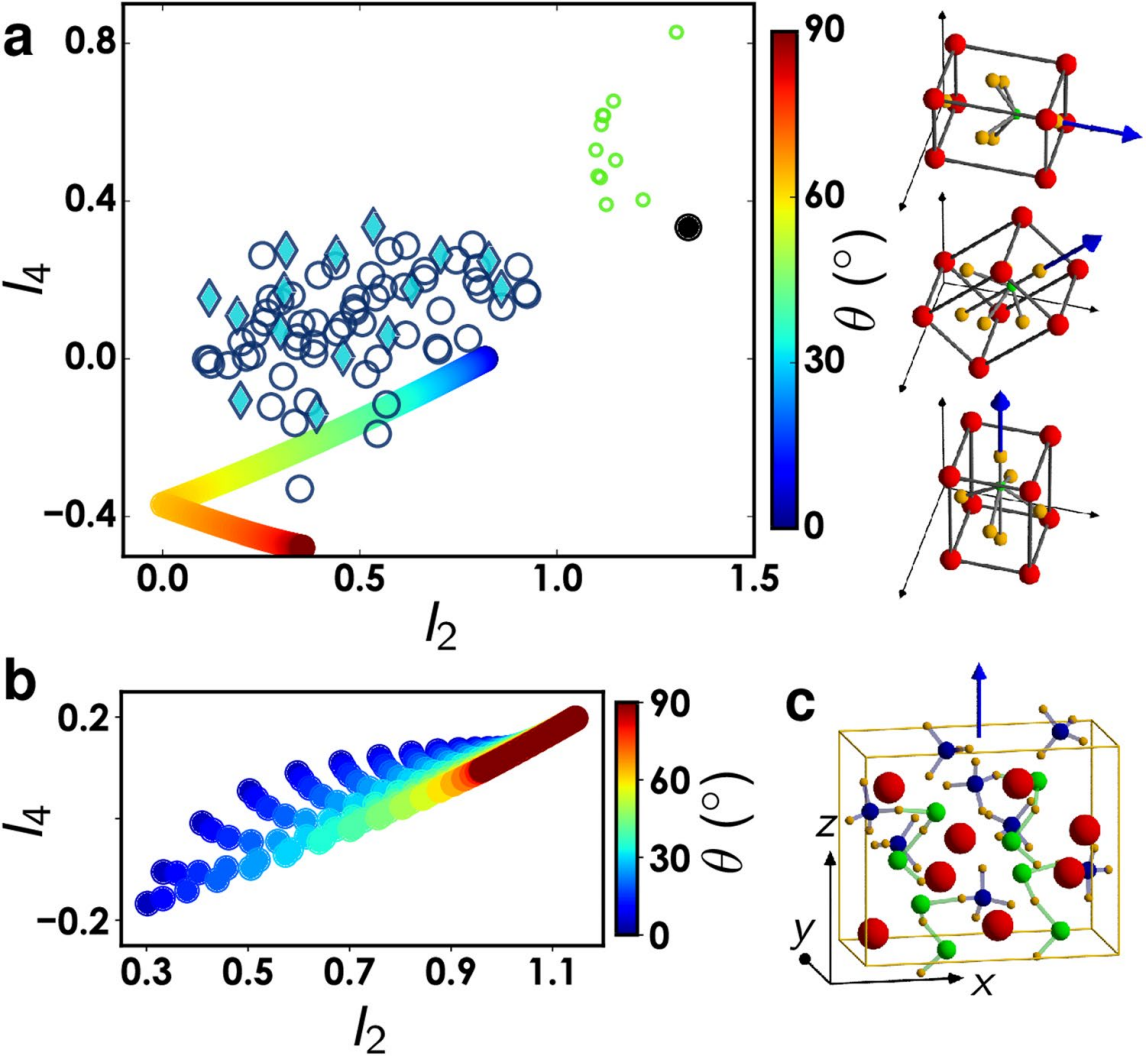
(**a**) *I*_2_ and *I*_4_ values reported for all measured BTO nanocrystals (markers), together with simulated values (thick colored line) resulting from the model of a pure tetragonal bulk crystal oriented with a varying *θ* from ~0° to 90° (the data are similar for all *φ* and *ψ* values), encoded as a color scale. Open circles indicate data for BTO nanocrystals in air, taking the averaged values over the central 25 pixels of the *I*_2_ pattern. Filled diamonds are similar data taken in oil. Small open green circles indicate values obtained on BTO nanocrystals in air, averaging the 25 pixels obtained at the border of the radial *I*_2_ pattern. The black dot indicates the response for a single isolated 1D dipole (i.e. ${\chi }_{xxx}^{(2)}=1$). BTO crystal unit cells oriented with *θ* = 90°, 45°, 0° are schematically represented. (**b**) *I*_2_ and *I*_4_ values for a simulated pure KTP crystal. (**c**) Illustration of a KTP crystal unit cell with the blue arrow indicating the main optical axis of the crystal. The atoms in the KTP unit cell illustration are colored red for potassium, green for titanium, blue for phosphorus and orange for oxygen.

While the in-plane *φ* orientation angle has an effect on the in-plane orientation of P-SHG patterns, the out-of-plane *θ* orientation has an effect on the shape of this pattern. A complementary read-out of this angle can be done via the measured P-SHG amplitudes *I*_2_ and *I*_4_, which are directly related to the unit-cell crystal symmetry and orientation. Assuming a bulk BTO crystal of tetragonal symmetry, the (*I*_2_, *I*_4_) values follow a specific dependence that is unique to such symmetry with variations due to the *θ* orientation of the unit cell relative to the longitudinal axis (Fig. 6a). The theoretical (*I*_2_, *I*_4_) dependence was then compared to the experimental obtained values, either from BTO nanocrystals in oil or in air. First, averages over all measured pixels in P-SHG maps show that despite the existence of the shell in the observed nanocrystals in air, the nonlinear anisotropy values obtained are close to that of a tetragonal symmetry as expected from the core crystalline structure (Fig. 6a). A slight offset is visible in *I*_4_ values, which we attribute to the presence of the surface response and the contribution from out-of-plane longitudinal coupling that are not accounted for here. Second, (*I*_2_, *I*_4_) values obtained at the contour of radial-like patterns leads to high *I*_2_ values that resemble pure dipolar responses (Fig. 6a). This result is also consistent with the one-dimensional nature of the surface response of the BTP nanocrystals in air.

The (*I*_2_, *I*_4_) dependence obtained for the BTO unit cell symmetry can be compared to that of a pure KTP crystal (Fig. 6b). Modelled (*I*_2_, *I*_4_) dependence for KTP was generated using its reported nonlinear coefficients^30^ and orientations defined according to its crystallographic data (Fig. 6c)^26^. A KTP crystal is seen to follow very different behavior from BTO (Fig. 6b), with a more pronounced dependence to all 3D Euler orientation angles. Overall the (*I*_2_, *I*_4_) dependence is thus the fingerprint of a bulk crystal symmetry as read-out by SHG, allowing possible unambiguous determination of its 3D orientations. It does not however reveal possible nanocrystal spatial heterogeneities, for which the previous analysis of the P-SHG response map is required.

To conclude, we have evidenced in single BTO nanocrystals the existence of a heterogeneous crystalline structure, which had been hypothesized by averaged structural analysis but was impossible to investigate from traditional polarization SHG approaches. This approach opens prospective for the *in situ* characterization of nonlinear nano-objects and the understanding of structural rules that govern nanomaterials fabrication, without X-rays. Importantly, in contrast to other structural analyzes methods such as high resolution transmission electron microscopy, this method offers observations over large field of views, allowing straightforward statistical explorations over many single nanocrystals, without requiring special substrates. This approach finally opens interesting prospectives in nanophotonics, allowing complex spatial polarization control over nanometric scales. Heterogeneous nanostructures provide for this purpose a large spatial variety of local polarization responses, which can be advantageously exploited in conjunction with polarized nonlinear optics.

## Methods

### Setup description

The setup is based on a two-photon scanning microscope which uses a Ti:Sapphire femtosecond laser and optical parametric oscillator (OPO) combination (150 fs, 80 MHz), at excitation wavelength in the range 800–1500 nm (Coherent Inc.). The total power delivered to the sample lied in the range of 1.0–1.2 mW. The laser beams sizes were expanded to fill the back aperture of the objective 40×/1.15 water immersion Nikon Instruments Inc.). Imaging was performed on an inverted microscope (Eclipse Ti, Nikon Instruments Inc.) using a pair of galvanometric scan mirrors (6215 H, Cambridge Technology Inc.). The transverse optical resolution is estimated to be 250 nm. The pixel size is 50 nm (field of view 5×5*μ*m, 100×100 pixels). A polarized beam splitter (PBS252, Thorlabs Inc.) was used to make the excitation lasers linearly polarized after the scan mirrors. This ensured that the dichroic mirror (T770SPXR, AHF Analysentechnik AG) received a p-polarized laser light during the entire experiment. After the dichroic mirror, the linear polarization angle of the excitation laser was controlled by an achromatic half-wave plate (AHWP10M-980, Thorlabs Inc.) mounted on a motorized rotational mount (PR50CC, Newport Corp.).

A stack of images was acquired by changing the polarization angle of the lasers in steps of 10° over the range of 0°–170°. The nonlinear signal collected by the objective was filtered using a shortpass filter (ET750sp-2p8, Chroma Technology Corp.) before being detected by a Photomultiplier tube (R9110, Hamamatsu Photonics K. K.). Depending on the excitation wavelength, the SHG signal was separated using an appropriate dichroic beamsplitter of 400/40 nm. The signal detection path consisted of imaging pairs of lenses which image the back aperture of the objective on to the detector window. This configuration keeps the signal steady on the detector even though the excitation spot is continuously scanning the sample. Scanning and data acquisition was performed using an in-house LabVIEW (National Instruments Corp.) program. The data is acquired by a data acquisition board (NI USB 6353, National Instruments Corp). Data analysis is performed using Matlab (The MathWorks, Inc).

### Data analysis

For drift correction, the images are first denoised (by a Gaussian filter) and the centroid of each nanoparticle image is calculated with pixel size accuracy (multiple nanoparticles within the same field of view can be tracked). Sometimes, the centroid cannot be automatically detected (in case of weak intensity peak that does not exceed a manual threshold of 25% of the maximum of the field of view) and the center of the peak is manually chosen. The obtained drift information is used to register all nanoparticle images in each polarization stack. Since the measurement is repeated (8 times), the drift-corrected nanoparticle stacks can be averaged to increase signal-to-noise which benefits the Fourier-analysis in P-SHG.

### BTO nanocrystals samples preparation

The BaTiO_3_ (BTO) nanocrystals used in this study are commercially available in the form of a powder from Techpowder S.A. and Nanostructured & Amorphous Materials, Inc. All particles have an average diameter of either about 100 nm or 300 nm. The size of the nanocrystal stated in this work is deduced from Dynamic Light Scattering experiments, which provide an average with a standard deviation depending on the sample. To give an indication, one batch of used BTO nanoparticles had a size distribution of 137 nm in average and a full width half maximum (FWHM) of 69 nm. The methodology used to prepare the BTO samples is straightforward. A stock suspension of nanoparticles in ethanol (1.2 mg to 1 mL) is sonicated (30 min). For a sample, 1 μL of the stock was diluted by 1:100 and a drop of 10 μL is deposited on a clean cover slip, which is then placed in vacuum spin coater (at 100 RPM for 9 s and 2000 RPM for 30 s) to dry the suspension and immobilize the nanoparticles.

### BTO Nanocrystals characterization: SEM imaging

The BTO nanoparticles are deposited on a ITO covered cover slip to avoid charging during scanning electron microscope (SEM) measurements. High resolution SEM micrographs are obtained in vacuum (<10^−5^ mbar) in a FEI Magellan 400 system (HV 2 kV, current 25 pA).

### KTP nanocrystals sample preparation

Potassium titanyl phosphate (KTP) is an efficient nonlinear optical material, usually grown to bulk crystals of macroscopic dimensions, but for which nanocrystals can be obtained with stable, high SHG radiation efficiency. In this work we use KTP nanocrystals fabricated by a co-precipitation method^42^. This permits to obtain stable KTP nanocrystals by colloidal synthesis in an aqueous solution, with excellent crystallinity and controllable size (100–150 nm). A diluted KTP-water solution is sonicated and a droplet placed onto a cleaned cover slip, previously cleaned and set in a UV-Ozone bath to make it hydrophobic.

## Supplementary information

Supplementary information
