# Supplementary material for: Crystalline heterogeneity in single ferroelectric nanocrystals revealed by polarized nonlinear microscopy
Source: Sci Rep. 2019 Feb 8;9:1670. doi: 10.1038/s41598-018-38229-4 (PMC6368600; doi:10.1038/s41598-018-38229-4)
Supplement: Supplementary file 1 — Supplementary information [file 41598_2018_38229_MOESM1_ESM.pdf]

Supplementary Information

Crystalline heterogeneity in single ferroelectric nanocrystals revealed by polarized nonlinear microscopy

Carolina Rendón-Barraza [1,§], Flavia Timpu [2], Rachel Grange [2], Sophie Brasselet [1,]*

[1] Aix Marseille Univ, CNRS, Centrale Marseille, Institut Fresnel, F-13013 Marseille, France.

[§] Present address : Ultrafast and Microspectroscopy Laboratories and ARC Centre of Excellence in Exciton Science, School of Chemistry, University of Melbourne, 3010, Australia

[2] Optical Nanomaterial Group, Institute for Quantum Electronics, Department of Physics, ETH Zurich, Auguste-Piccard-Hof 1, 8093 Zurich, Switzerland

[*] E-mail: Sophie.brasselet@fresnel.fr

Modelling P-SHG imaging in $BaTiO_3$ heterogeneous nanocrystals

To model the P-SHG imaging response of a spatially heterogeneous nonlinear nanocrystal, this nanocrystal is represented by a set of nonlinear induced dipoles placed within the crystal area (at positions r), each of them specific of the local nonlinear tensor representing the local unit cell. The model limits the approach to a 2D object, supposing that the other planes nonlinear dipoles contribute similarly and thus do not introduce drastic modifications. The induced nonlinear dipole at the position r, excited by the local field $E_{loc}^{\omega}(r)$ at the position r of the particle can be written:

$$p^{2\omega}(r) = \chi^{(2)}(r) \colon E_{loc}^{\omega}(r) E_{loc}^{\omega}(r)$$

Note that in what follows, the dipole and field quantities are vectors. We suppose that the incident field E^{ω} exhibits a homogeneous polarization within the sample plane, which is reasonable assumption in a first approximation as discussed below. This incident field, at the position r, can be written:

$$E^{\omega}(r)E^{\omega}(r) = E^{\omega}E^{\omega}\ G(r)$$

With $G(r)$ is a Gaussian function, which size is that of the focused field that scans the sample. The size of this Gaussian function corresponds to the resolution of a two photon microscope. When the spot is parked at the position r_0 it writes:

$$G(r) = Gauss(r - r_0)$$

The nonlinear tensor is spatially distributed within the particle following its structural properties. For the considered BTO crystal structure of size (diameter) s, made of a tetragonal core of diameter c, a cubic shell, and a surface of small size ε, the corresponding nonlinear tensor can be modelled as:

$$\chi^{(2)}(r) = \chi^{(2)}_{BTO} \quad \text{for} \quad 0 < r < \frac{c}{2}$$

$$\chi^{(2)}(r) = 0 \quad \text{for} \quad \frac{c}{2} < r < \frac{s}{2}$$

$$\chi^{(2)}(r) = \chi^{(2)}_{nnn}(r) \quad \text{for} \quad \frac{s}{2} - \varepsilon < r < \frac{s}{2}$$

With $\chi_{BTO}^{(2)} = \begin{bmatrix} 0 & 0 & 0 & 0 & \chi_{223}^{(2)} & 0 \\ 0 & 0 & 0 & \chi_{113}^{(2)} & 0 & 0 \\ \chi_{311}^{(2)} & \chi_{322}^{(2)} & \chi_{333}^{(2)} & 0 & 0 & 0 \end{bmatrix}$ in the BTO crystal frame. The nonlinear coefficients used in this work for BTO are $\chi_{223}^{(2)} = \chi_{232}^{(2)} = \chi_{113}^{(2)} = \chi_{131}^{(2)} = 38.7\, pm/V$, $\chi_{322}^{(2)} = \chi_{311}^{(2)} = 35.7\, pm/V, \chi_{333}^{(2)} = 15.6\, pm/V$[1,2].

The surface contribution $\chi_{nnn}^{(2)}$ points in the normal direction of the nanocrystal surface (here supposed as a circle) at the considered position r, which defines locally the direction n. The magnitude of the surface contribution is scales by a factor η such as $\chi_{nnn}^{(2)} = \eta . \left\| \chi_{BTO}^{(2)} \right\|$ with $\left\| \chi_{BTO}^{(2)} \right\| = \sqrt{\sum_{i,j,k} \left(\chi_{ijk}^{(2)} \right)^2}$ the norm of the BTO nonlinear tensor.

At last, to account for the 3D orientation of the nanocrystal in the (X, Y, Z) macroscopic coordinates, we apply a rotation transformation that expresses the tensor $\chi^{(2)}(r)$ in the macroscopic frame with components $\chi_{IJK}^{(2)}$ (with $(I, J, K) = X, Y$). Those macroscopic components are related to the unit-cell components $\chi_{ijk}^{(2)}$ ($(i, j, k) = 1,2,3$) by:

$$\chi_{IJK}^{(2)} = \sum_{i,j,k} \chi_{ijk}^{(2)} (i.I)(j.J)(k.K)$$

With $(i.I) = R_{iI}$ the projection of the i axis on the I axis accounting for the transformation matrix:

$$R = \begin{bmatrix} \cos\theta\cos\varphi\cos\psi - \sin\varphi\sin\psi & -\cos\theta\cos\varphi\sin\psi - \sin\varphi\cos\psi & \cos\varphi\sin\theta \\ \cos\theta\sin\varphi\cos\psi + \cos\varphi\sin\psi & -\cos\theta\sin\varphi\sin\psi + \cos\varphi\cos\psi & \sin\varphi\sin\theta \\ -\sin\theta\cos\psi & \sin\theta\sin\psi & \cos\theta \end{bmatrix}$$

With (θ, φ, ψ) the Euler set of angle defining the 3D orientation of the nanocrystal in the sample frame.

Since the local field polarization inside the particle follows the incident polarization direction with a homogeneous magnitude (see Figure S2 below), the local field directions considered for the SHG polarization responses will thus follow $E^{\omega}(r)$ inside the particle:

$$p^{2\omega}(r < \frac{s}{2} - \varepsilon) \propto \chi^{(2)}(r): E^{\omega}(r)E^{\omega}(r)$$

At its surface, the local field is normal to the particle contour (Figure S2), therefore:

$$p^{2\omega}(\frac{s}{2} - \varepsilon < r < \frac{s}{2}) = \chi^{(2)}_{nnn}(r): E^{\omega}_{n}(r)E^{\omega}_{n}(r)$$

At last, to calculate the P-SHG response recorded for a given park position r_0 of the incident focused beam, the sample plane is discretized in pixels of size smaller than the diffraction limit (here typically 40 nm, the size of the focal spot being 250 nm). Each pixel-dipole radiates through the imaging system made of the microscope objective and the tube lens, with a radiated field written:

$$E^{2\omega}(k, r) \propto k \times k \times p^{2\omega}(r)$$

In the paraxial approximation, which is used here in a first approximation which is reasonable for the NA used, the k vector is considered close to the optical axis and the field can be simplified into a longitudinal-propagating field: $E^{2\omega}(r) \propto p^{2\omega}(r)$. When considering many dipoles covered by the excitation spot, the total field is the coherent sum of fields for all radiating dipoles present in this focal spot:

$$E^{2\omega} \propto \int_{r \in PSF} E^{2\omega}(r) dr$$

The measured intensity is finally: $I^{2\omega} \propto |E^{2\omega}|^2$.

Note that if all dipoles are identical within the focal spot, the total intensity is proportional to the intensity of a single dipole, which permits to model the nonlinear signal build up by an incoherent sum that avoids heavy calculation steps. We have noticed that such incoherent sum, for the crystal structure considered, is equivalent to the coherent model described above for nanocrystal sizes above 100 nm.

Finally, the assumptions made in the model used in this work are the following:

- The incident polarization is homogeneous in the focal plane, and does not contain longitudinal contributions. This assumption ignores in particular the possible effects of high NA focusing, which distributes the field polarization over different components (including longitudinal), especially at the edge of the focal plane and for NA > 1. This assumption has been shown to be valid as long as the crystal main axis is not highly tilted out of plane[3]. Deviations, that are often small in the majority of cases investigated in this work, can however be modelled by inserting a rigorous expression of the incident field in the nonlinear dipole expression (see below).

- The emission polarization is written in the planar wave approximation, which is in principle valid only at low NA. Rigorously, the fields should be summed up over all the radiation pattern of the dipoles: $E^{2\omega} \propto \int_{r \in PSF} \int_{k \in NA} E^{2\omega}(k, r) dr dk$. This assumption ignores in particular possible polarization mixtures occurring at high NA collection. This effect

however can be ignored when no analyzer is placed at the detection, which is then insensitive to polarization re-distributions.

- The crystal is placed in a homogeneous medium, therefore we ignore the effect of the glass substrate surface which can affect the emission polarization state. Here again since we do not place any analyzer at the detection step, this effect can be neglected since it contributes majorly to re-distribute emission polarizations.

References

1. S. Singh. *Handbook of Lasers With Selected Data on Optical Technology by Pressley, Robert J. (Editor): The Chemical Rubber Co. Hardcover - BookDepart*. (The Chemical Rubber Co., 1971).
2. Miller, R. C. Optical harmonic generation in single crystal BaTiO3. *Phys. Rev.* (1964). doi:10.1103/PhysRev.134.A1313
3. Schön, P., Behrndt, M., Aït-Belkacem, D., Rigneault, H. & Brasselet, S. Polarization and phase pulse shaping applied to structural contrast in nonlinear microscopy imaging. *Phys. Rev. A* **81,** 13809 (2010).

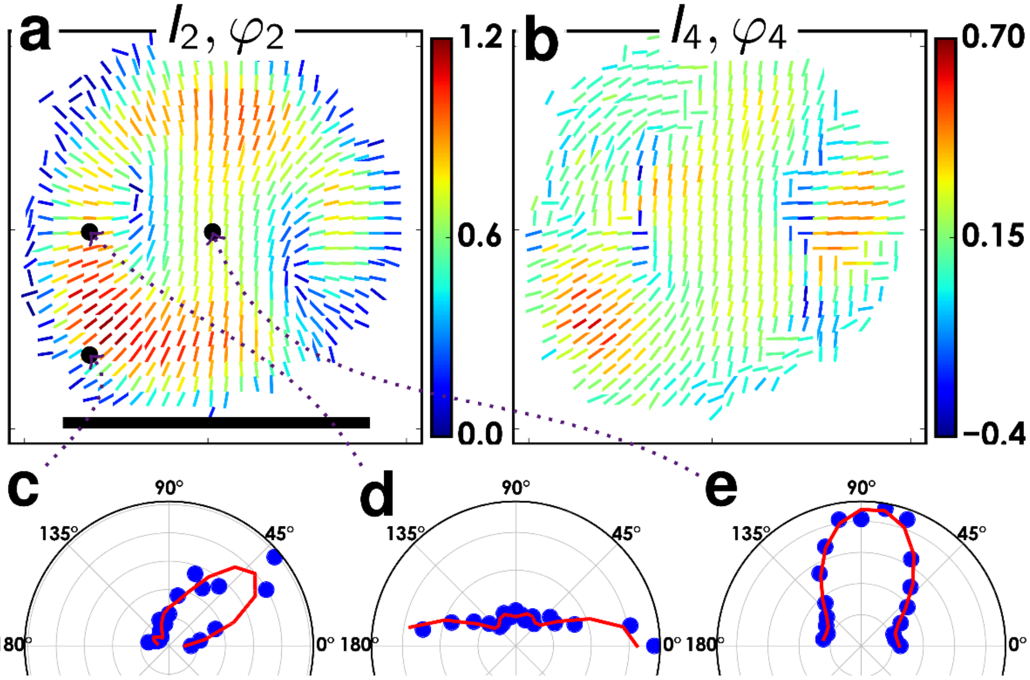

Figure S1. Experimental P-SHG maps and local responses measured for a single isolated BTO nanocrystal. (a) (I_2, φ_2) image. (b) (I_4, φ_4) image. (c-e) SHG intensity as a function of polarization (α) for three pixel locations, showing experimental data (markers) and Fourier decomposition up to the sixth order (line). Scale bar: 1000 nm.

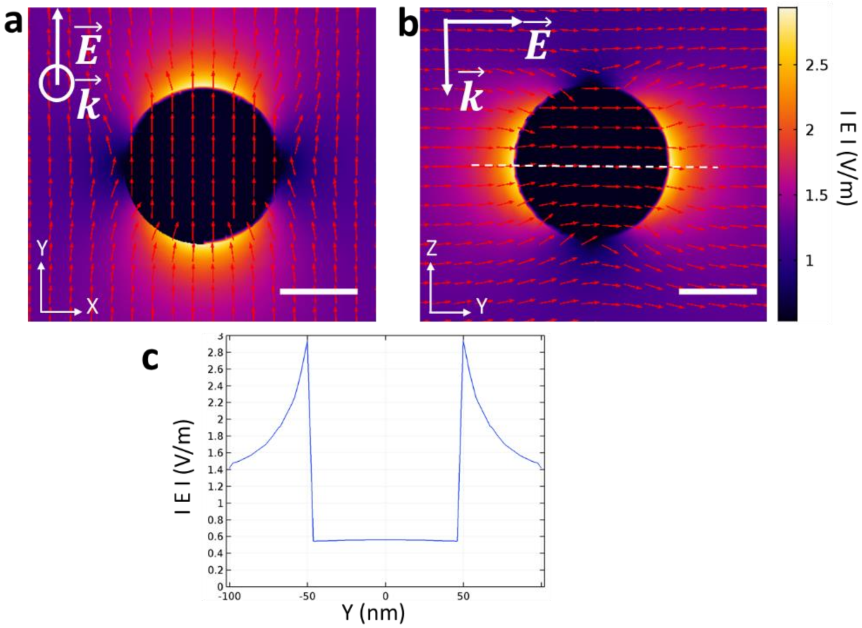

Figure S2. Simulated maps of the field scattered by a 100 nm size $BaTiO_3$ nanocrystal. The scale is the field amplitude, and the red arrows show the field polarization for incoming polarization along Y. (a) Projection in the sample plane XY. (b) Projection in the incidence plane XZ, with Z the propagation direction. (c) Profile of the field amplitude along the dashed line shown in (b). The electric field distribution is calculated using a finite element method (FEM) software (COMSOL Multiphysics 5.3). A 100 nm diameter spherical $BaTiO_3$ nanoparticle is positioned on a glass substrate. The incident field reaches the air-glass surface at normal incidence, and the background field is calculated using the Fresnel coefficients before being solved for the scattered field. The total electric field is the sum between the scattered field and the background. The values of the refractive index of the BTO and the glass are taken from the literature [Wong, C., Teng, Y. Y., Ashok, J. & Varaprasad, P. L. H. Barium titanate (BaTiO3). Handb. Opt. Constants Solids II 789–803 (1991) ; Malitson, I. H. Interspecimen Comparison of the Refractive Index of Fused Silica. JOSA 55, 1205–1209 (1965)]. Scale bars: 50 nm.

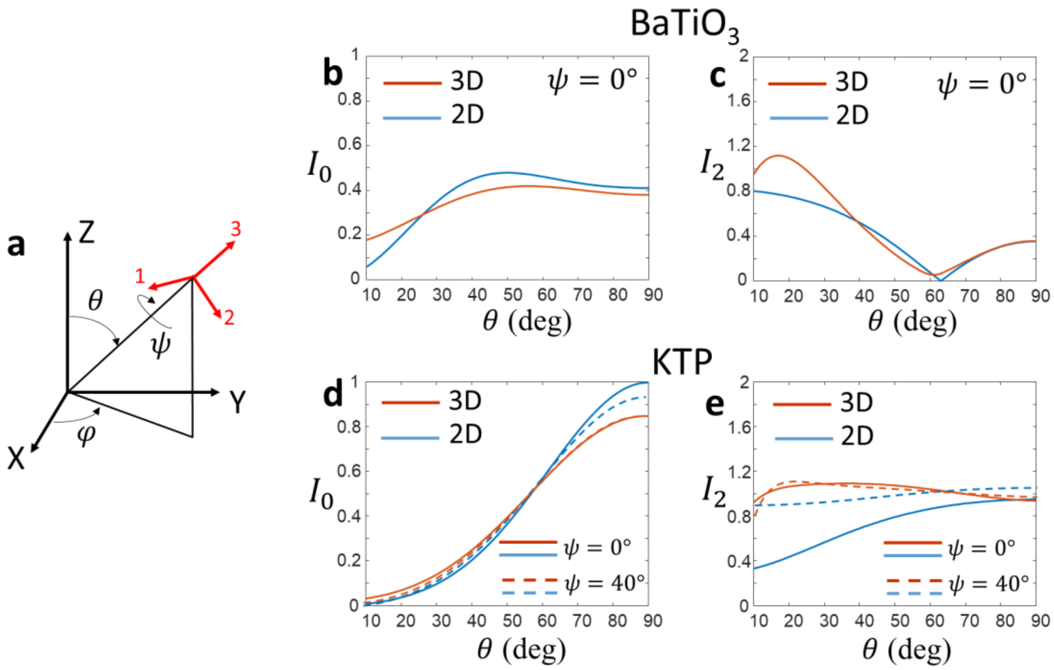

Figure S3. Theoretical P-SHG values obtained for bulk model crystals in 3D. (a) The crystals are oriented using the Euler angles $(\theta, \varphi = 0°, \psi)$ and their nonlinear coefficient defined in their (1,2,3) unit-cell frame. Two crystals are studied: $BaTiO_3$ bulk tetragonal symmetry (b,c) and KTP (d,e). For each crystal, the total intensity I_0 is given as a function of θ (b,d) and the anisotropy P-SHG value intensity I_2 is given similarly as a function of θ (c,e). For KTP, two angles ψ are depicted since the 3D behavior of the crystal depends on ψ, which is not the case for $BaTiO_3$. The calculation accounts for a longitudinal contribution of the incoming polarization, modelled as an additional component of the incident polarization along the propagation direction Z, writing $\boldsymbol{E}(\alpha) = (E_X.cos\alpha, E_Y.sin\alpha, E_Z)$. The magnitude of this longitudinal contribution scales as 33% times the in-plane contributions. This value is obtained by averaging the E_Z contribution over the focused field point spread function, calculated at NA = 1.15, using [Richards, B. & Wolf, E. Electromagnetic Diffraction in Optical Systems. II. Structure of the Image Field in an Aplanatic System. Proc. R. Soc. A Math. Phys. Eng. Sci. 253, 358–379 (1959)].

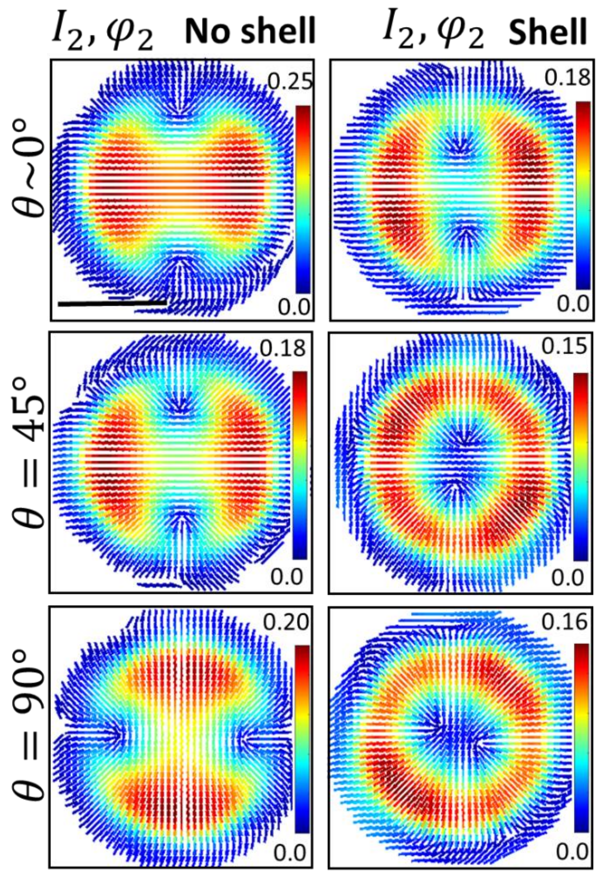

Figure S4. Theoretical P-SHG images accounting for a longitudinal contribution of the incoming polarization, for a 100 nm size nanocrystal, at different θ angles for a pure crystal (left) and in presence of the shell (right, using c/s = 0.4, surface-to-core nonlinear efficiency ratio η = 0.5). The field longitudinal contribution is considered as an additional homogeneous component of incident polarization along the propagation direction Z, similarly as in Figure S3. The model does not account for the spatial repartition of E_Z, which is in reality stronger at the border of the point spread function and negligible at its center. The averaged calculation used here gives nevertheless an upper limit of the effect of E_Z on P-SHG maps. Scale bar: 500 nm.

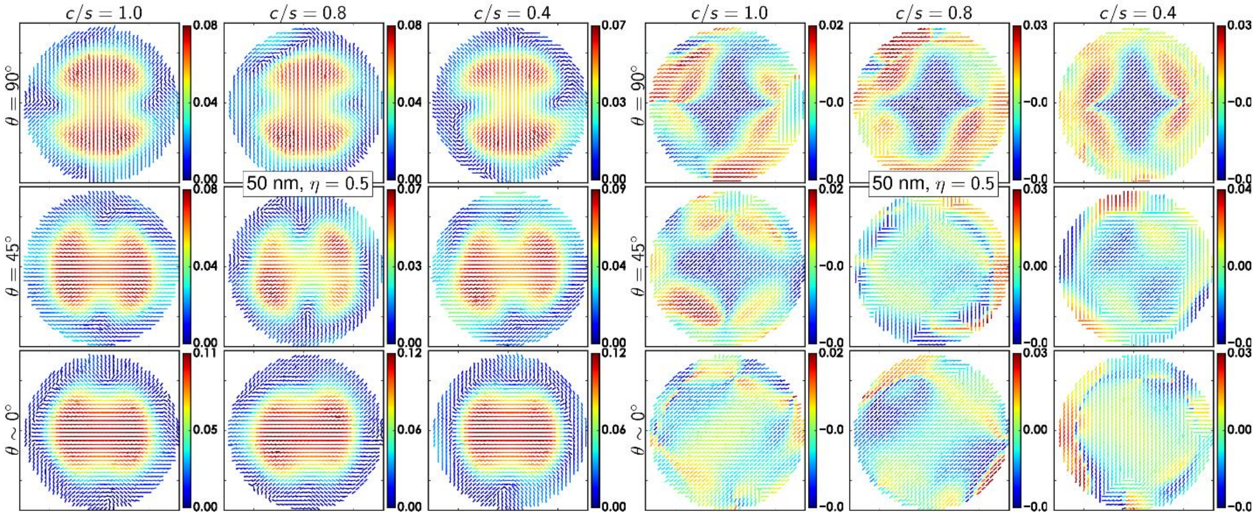

Figure S5: Modeled (I_2, φ_2) maps (Left) and (I_4, φ_4) maps (Right) for varying core-to-size ratios c/s and $\boldsymbol{s} = \mathbf{50\ nm}$, $\varepsilon = 40$ nm, $\boldsymbol{\eta} = \mathbf{0.5}$. Pixel size : 40 nm.

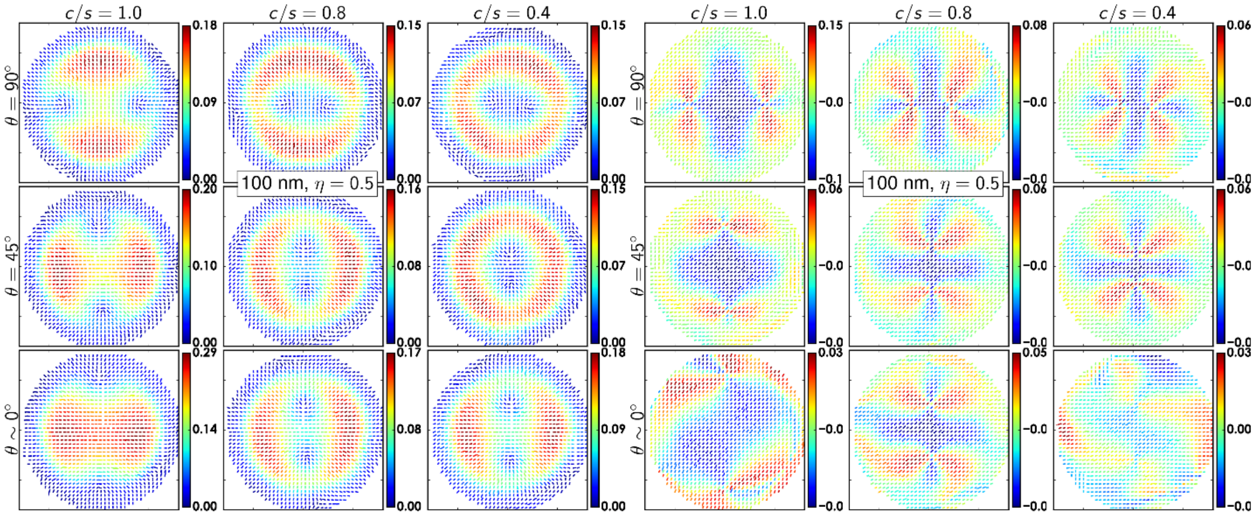

Figure S6: Modeled (I_2, φ_2) maps (Left) and (I_4, φ_4) maps (Right) for varying core-to-size ratios c/s and $\boldsymbol{s} = \mathbf{100\ nm}$, $\varepsilon = 40$ nm, $\boldsymbol{\eta} = \mathbf{0.5}$. Pixel size : 40 nm.

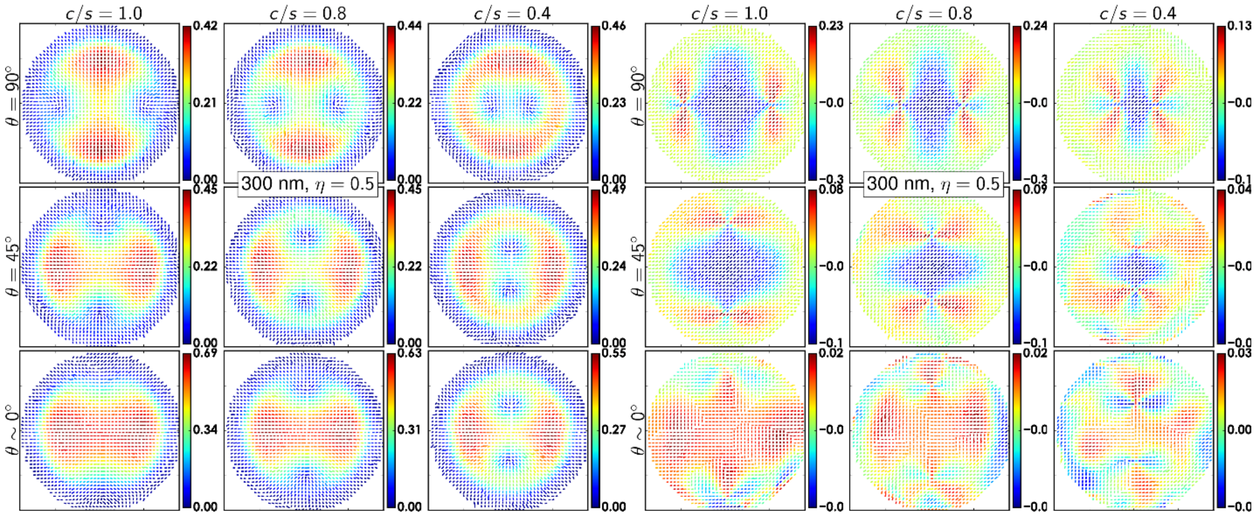

Figure S7: Modeled (I_2, φ_2) maps (Left) and (I_4, φ_4) maps (Right) for varying core-to-size ratios c/s and $\boldsymbol{s} = \mathbf{300\ nm}$, $\varepsilon = 40$ nm, $\boldsymbol{\eta} = \mathbf{0.5}$. Pixel size : 40 nm.

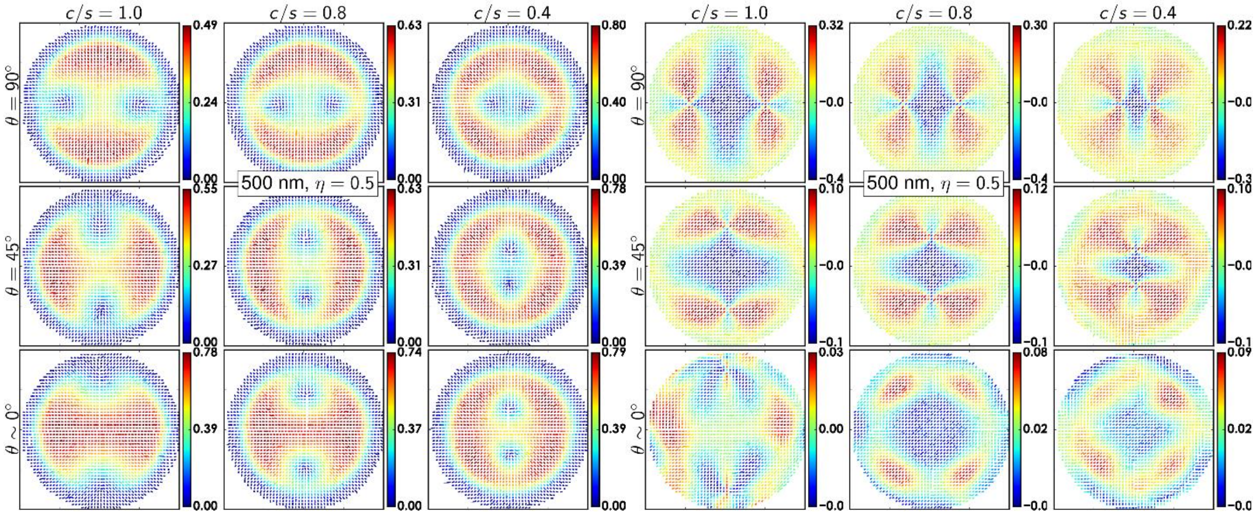

Figure S8: Modeled (I_2, φ_2) maps (Left) and (I_4, φ_4) maps (Right) for varying core-to-size ratios c/s and $\boldsymbol{s} = \mathbf{500\ nm}$, $\varepsilon = 40$ nm, $\boldsymbol{\eta} = \mathbf{0.5}$. Pixel size : 40 nm.

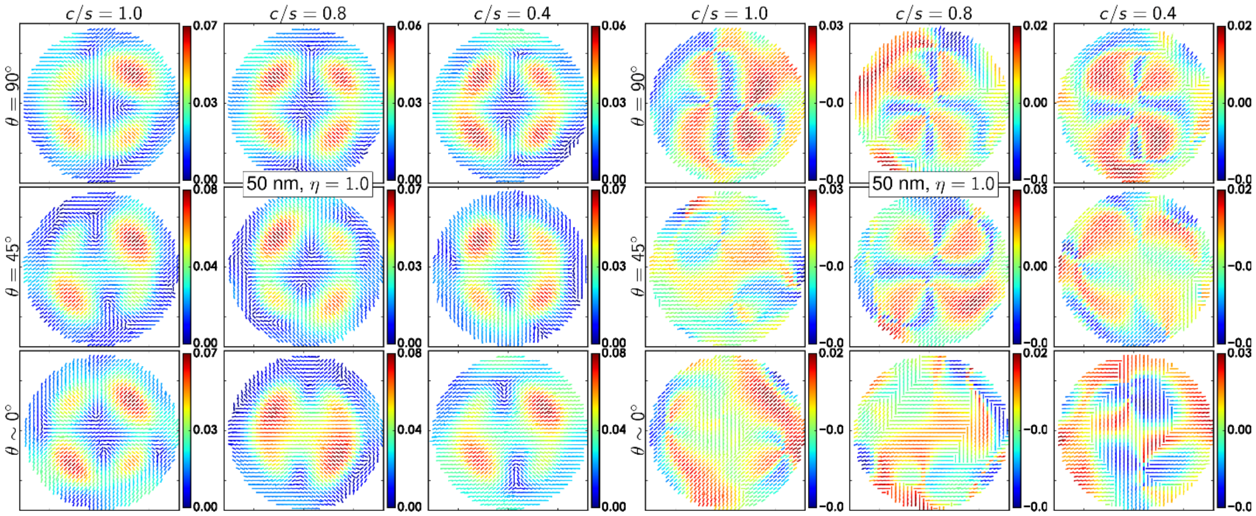

Figure S9: Modeled (I_2, φ_2) maps (Left) and (I_4, φ_4) maps (Right for varying core-to-size ratios c/s and $\boldsymbol{s} = \mathbf{50\ nm}$, $\varepsilon = 40$ nm, $\boldsymbol{\eta} = \mathbf{1}$. Pixel size : 40 nm.

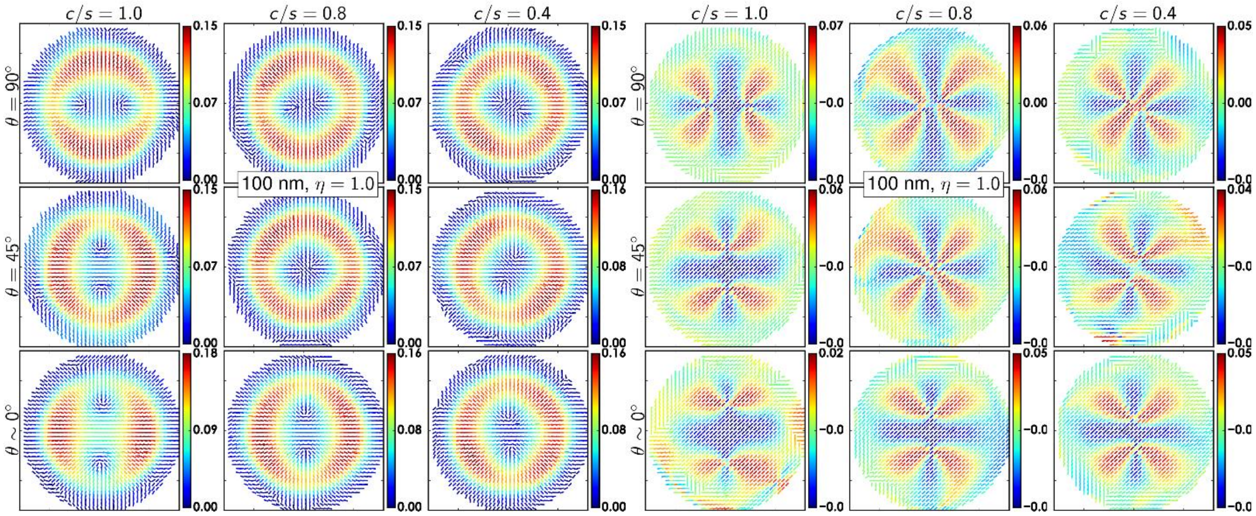

Figure S10: Modeled (I_2, φ_2) maps (Left) and (I_4, φ_4) maps (Right) for varying for varying core-to-size ratios c/s and $\boldsymbol{s} = \mathbf{100\ nm}$, $\varepsilon = 40$ nm, $\boldsymbol{\eta} = \mathbf{1}$. Pixel size : 40 nm.

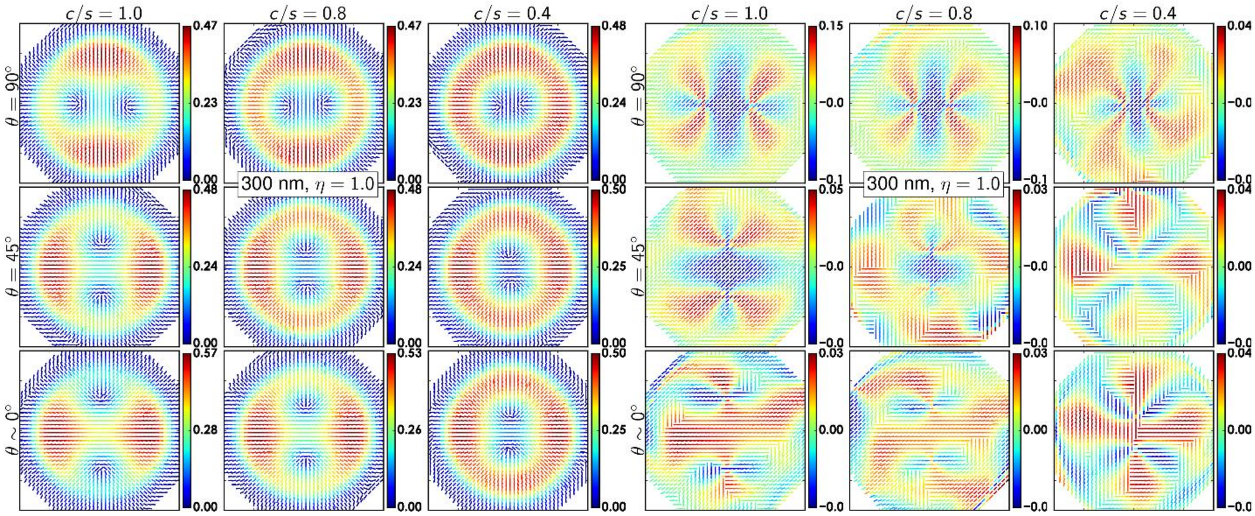

Figure S11: Modeled (I_2, φ_2) maps (Left) and (I_4, φ_4) maps (Right) for varying core-to-size ratios c/s and $\boldsymbol{s} = \mathbf{300\ nm}$, $\varepsilon = 40$ nm, $\boldsymbol{\eta} = \mathbf{1}$. Pixel size : 40 nm.

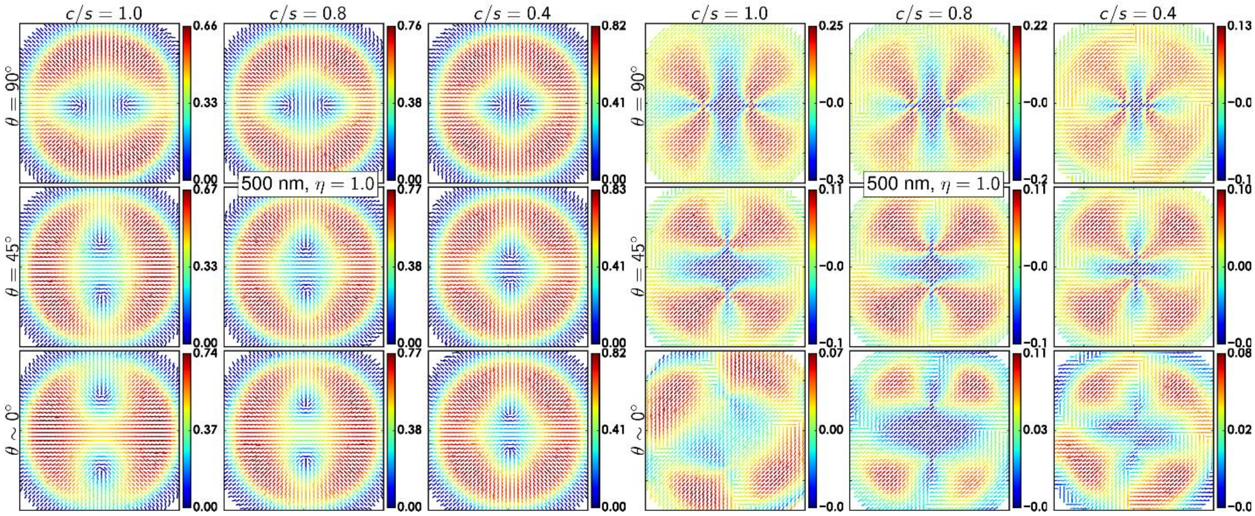

Figure S12: Modeled (I_2, φ_2) maps (Left) and (I_4, φ_4) maps (Right) for varying core-to-size ratios c/s and $\boldsymbol{s} = \mathbf{500\ nm}$, $\varepsilon = 40$ nm, $\boldsymbol{\eta} = \mathbf{1}$. Pixel size : 40 nm.

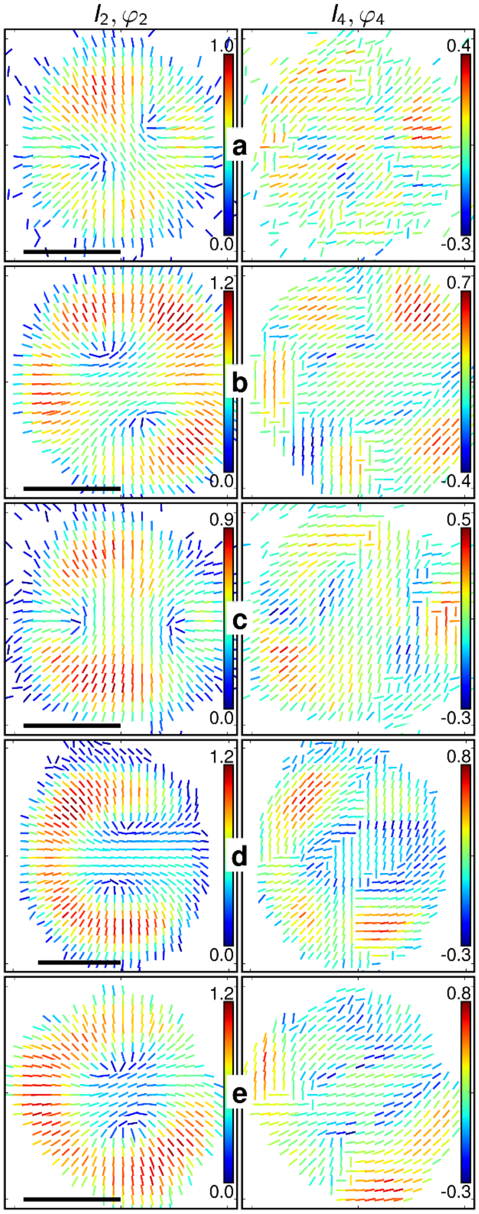

Figure S13: (a-e) Examples of P-SHG experimental results obtained for BTO nanocrystals (expected size 100 nm) for which the obtained (I_2, φ_2) patterns slightly departs from a radial shape. The results resemble, for most of them, theoretical patterns for which the particle size is about 100 nm, with orientations close to $\theta < 45°$, core-to-size ratio of $c/s = 0.4$, and $\eta = 0.5$. Scale bars: 500 nm.

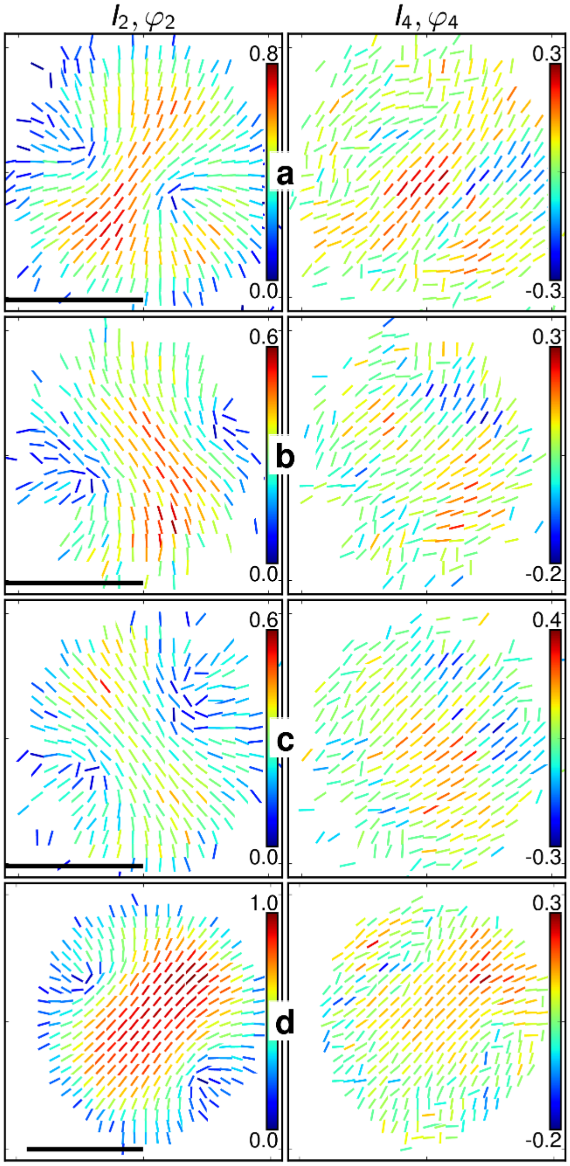

Figure S14: (a-d) Examples of P-SHG experimental results obtained for BTO nanocrystals (expected size 100 nm) for which the obtained (I_2, φ_2) patterns strongly departs from a radial shape. The results resemble, for most of them, theoretical patterns for which the particle size is about 300 nm, with orientations close to $\theta < 45°$, core-to-size ratio of $c/s = 0.4$ to 0.8, and $\eta = 0.5$. Scale bars: 500 nm.
